# Targeted sequencing identifies genetic alterations that confer primary resistance to EGFR tyrosine kinase inhibitor (Korean Lung Cancer Consortium)

**DOI:** 10.18632/oncotarget.8904

**Published:** 2016-04-21

**Authors:** Sun Min Lim, Hye Ryun Kim, Eun Kyung Cho, Young Joo Min, Jin Seok Ahn, Myung-Ju Ahn, Keunchil Park, Byoung Chul Cho, Ji-Hyun Lee, Hye Cheol Jeong, Eun Kyung Kim, Joo-Hang Kim

**Affiliations:** ^1^ Department of Internal Medicine, Division of Medical Oncology, Yonsei Cancer Center, Yonsei University College of Medicine, Seoul, Korea; ^2^ Department of Internal Medicine, Division of Medical Oncology, CHA Bundang Medical Center, CHA University, Seongnam, Korea; ^3^ Department of Internal Medicine, Gachon University Gil Medical Center, Incheon, Korea; ^4^ Department of Oncology, Asan Medical Center, University of Ulsan college of Medicine, Seoul, Korea; ^5^ Division of Hematology-Oncology, Department of Medicine, Samsung Medical Center, Sungkyunkwan University School of Medicine, Seoul, Korea; ^6^ Department of Internal Medicine, CHA Bundang Medical Center, CHA University, Seongnam, Korea

**Keywords:** primary resistance, epidermal growth factor, next-generation sequencing, tyrosine kinase inhibitor

## Abstract

**Background:**

Non-small-cell lung cancer (NSCLC) patients with activating epidermal growth factor receptor (*EGFR*) mutations may exhibit primary resistance to EGFR tyrosine kinase inhibitor (TKI). We aimed to examine genomic alterations associated with *de novo* resistance to gefitinib in a prospective study of NSCLC patients.

**Methods:**

One-hundred and fifty two patients with activating *EGFR* mutations were included in this study and 136 patients' tumor sample were available for targeted sequencing of genomic alterations in 22 genes using the Colon and Lung Cancer panel (Ampliseq, Life Technologies).

**Results:**

All 132 patients with *EGFR* mutation were treated with gefitinib for their treatment of advanced NSCLC. Twenty patients showed primary resistance to EGFR TKI, and were classified as non-responders. A total of 543 somatic single-nucleotide variants (498 missense, 13 nonsense) and 32 frameshift insertions/deletions, with a median of 3 mutations per sample. *TP53* was most commonly mutated (47%) and mutations in *SMAD4* was also common (19%), as well as *DDR2* (16%), *PIK3CA* (15%), *STK11* (14%), and *BRAF* (7%). Genomic mutations in the *PI3K/Akt/mTOR* pathway were commonly found in non-responders (45%) compared to responders (27%), and they had significantly shorter progression-free survival and overall survival compared to patients without mutations (2.1 *vs*. 12.8 months, *P*=0.04, 15.7 *vs.* not reached, *P*<0.001). *FGFR* 1-3 alterations, *KRAS* mutations and *TP53* mutations were more commonly detected in non-responders compared to responders.

**Conclusion:**

Genomic mutations in the *PI3K/Akt/mTOR* pathway were commonly identified in non-responders and may confer resistance to EGFR TKI. Screening lung adenocarcinoma patients with clinical cancer gene test may aid in selecting out those who show primary resistance to EGFR TKI (NCT01697163).

## INTRODUCTION

Lung cancer is the leading cause of cancer deaths worldwide [1]. Adenocarcinoma, which consists of more than 50% of non-small-cell lung cancer (NSCLC), is the most frequent type. Recent efforts to characterize molecular subclassifications of NSCLC have provided a marked benefit to patients whose tumors harbor specific genetic alterations [2–4], and the three major driver oncogenic mutations are epidermal growth factor receptor (*EGFR*) mutation, *KRAS* mutation, and anaplastic lymphoma kinase (*ALK*) rearrangement.

*EGFR* activating mutations are the most important predictive markers of response to EGFR tyrosine kinase inhibitor (TKI) treatment [5]. Despite the demonstrated benefits of EGFR TKIs, not all patients respond to treatment. Approximately 30% of patients with *EGFR* activating mutations do not show objective responses to EGFR TKI [6]. Intrinsic, *de novo* or primary resistance is defined as the failure to respond to *EGFR*-targeted therapies and little is known about the mechanisms of primary resistance. On the contrary, acquired resistance occurs in patients who initially benefited from EGFR-targeted therapies and the underlying mechanisms of acquired resistance include *EGFR* T790M mutation, activation of bypass signaling (such as *MET* amplification, *HER2* upregulation or *KRAS* activation), and histologic transformation to small cell lung cancer or epithelial-mesenchymal transition [7].

Recent studies have revealed that both somatic mutations and germline polymorphisms may result in primary resistance to EGFR TKI. For example, mutations in phosphoinositide-3-kinase catalytic alpha (*PIK3CA*), the p110α catalytic subunit of phosphatidylinositol-3-kinase, are found in approximately 4% of NSCLC patients [8] and result in resistance to EGFR TKI. Loss of phosphatase and tensin homolog (*PTEN*) and *de novo MET* amplification could also be associated with resistance [9, 10]. In addition, germline polymorphisms of BIM, a pro-apoptotic protein, which result in *BIM* deletion may confer primary resistance [11]. SRC and MAP kinase pathways may also act as bypass pathways which confer resistance to EGFR TKIs [12]. However, other mechanisms of primary resistance remain largely unknown.

With the advancement of next-generation sequencing (NGS), it is now possible to identify oncogenic alterations that would previously been missed by conventional sequencing. Rather than sequencing the entire genome or exome, clinical cancer gene test which include genes that show frequent alterations in cancer can save the amount of tissue, time and effort to perform sequencing. These panels use PCR capture-based NGS assay that allow deep targeted sequencing of genes of interest from limited formalin-fixed, paraffin-embedded (FFPE) specimens [13]. Since incorporating NGS into routine oncologic practice requires accurate genomic profiling in a single assay, clinical cancer gene test may be appropriately used for clinical use.

In this study, we aimed to discover novel mechanisms of primary resistance to EGFR TKIs by using patient tumor samples from a large-scaled, prospective trial. We performed clinical cancer gene test of patient tissue samples which were obtained before treatment with EGFR TKIs in order to identify genetic alterations that confer primary resistance to EGFR TKIs.

## RESULTS

### Patient characteristics

The baseline characteristics of all patients are summarized in Table 1. The median age of all patients was 60 (range, 32-84) and there were 86 females (63.3%). The majority of patients (61%) were never-smokers and nearly all patients had adenocarcinoma histology (97.8%). At the time of their cancer diagnosis, 1 patient (0.7%) had stage IIIB disease, 119 (87.5%) had stage IV disease, and 16 (11.8%) had relapsed after surgical resection of lung cancer. *EGFR* mutations included exon 19 deletion (n=75), L858R mutation (n=65) and the rest included G719X, L861Q and others (n=6). Ten patients had two or more coexisting *EGFR* mutations (complex mutation).

**Table 1:** Baseline characteristics of all patients (N=136)

Characteristic	N	%
Age (years)		
Median	60	
Range	32-84	
Gender		
Male	50	36.7
Female	86	63.3
Smoking history		
Never-smoker	83	61
Ever smoker	53	49
Histologic diagnosis		
Adenocarcinoma	133	97.8
Squamous	1	0.7
Adenosquamous	2	1.5
Clinical stage		
IIIB	1	0.7
IV	119	87.5
Postoperative relapse	16	11.8
Type of *EGFR* mutation*		
Exon 19 deletion	75	51.4
L858R	65	44.5
Others*	6	4.1

* 10 patients had two or more coexsiting *EGFR* mutations

### Treatment outcome of EGFR TKI

**Figure 1:**
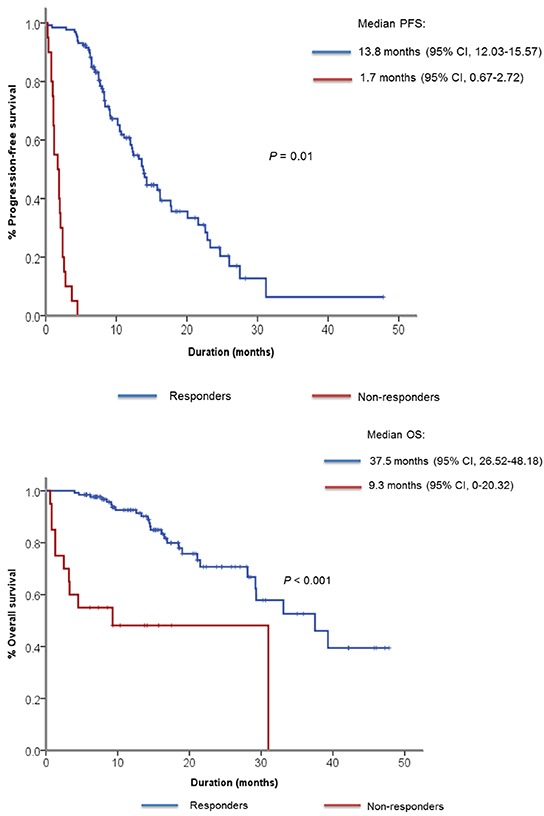
Kaplan-Meier curve showing **A.** median progression-free survival and **B.** median overall survival among responders and non-responders to gefitinib.

The median follow-up duration was 14 months and 101 (74.3%) patients received gefitinib as their first-line of treatment. As for best response, 87 patients (63.8%) showed partial response (PR), 33 patients (24.5%) showed SD and 6 patients (4.4%) showed PD (Table 2). Ten patients (7.3%) had not undergone response evaluation due to clinical disease progression, study withdrawal and follow-up loss. According to our prespecified definition of primary resistance to EGFR TKI, 20 patients showed PD as best response to gefitinib or PFS of less than 4 months. We classified them as non-responders to gefitinib. The median PFS was 9.1 months (95% confidence interval [CI] 7.15 – 11.05) for all patients, 13.8 months (95% CI, 12.03 – 15.57) for responders, 1.7 months (95% CI, 0.67 – 2.72) for non-responders (Figure 1A). The median OS for responders was 37.5 months (95% CI, 26.52 – 48.18), whereas it was 9.3 months (95% CI, 0.0-20.32) for non-responders (Figure 1B). When OS was compared only among patients who received gefitinib for their first line therapy, the median OS was 37.5 months (95% CI, 24.98 – 50.02) for responders and 4.5 months (95% CI, 0 – 16.19) for non-responders (Supplementary Figure S1).

**Table 2:** Summary of EGFR TKI treatment outcome

Characteristic	N	%
Number of previous treatment		
0	101	74.3
1	32	23.5
2	3	2.2
Best response		
Complete response	0	0
Partial response	87	63.8
Stable disease	33	24.5
Progressive disease	6	4.4
Not assessable	10	7.3
EGFR TKI		
Gefitinib	136	100
Erlotinib	0	0

### Genomic landscape of responders and non-responders

**Figure 2:**
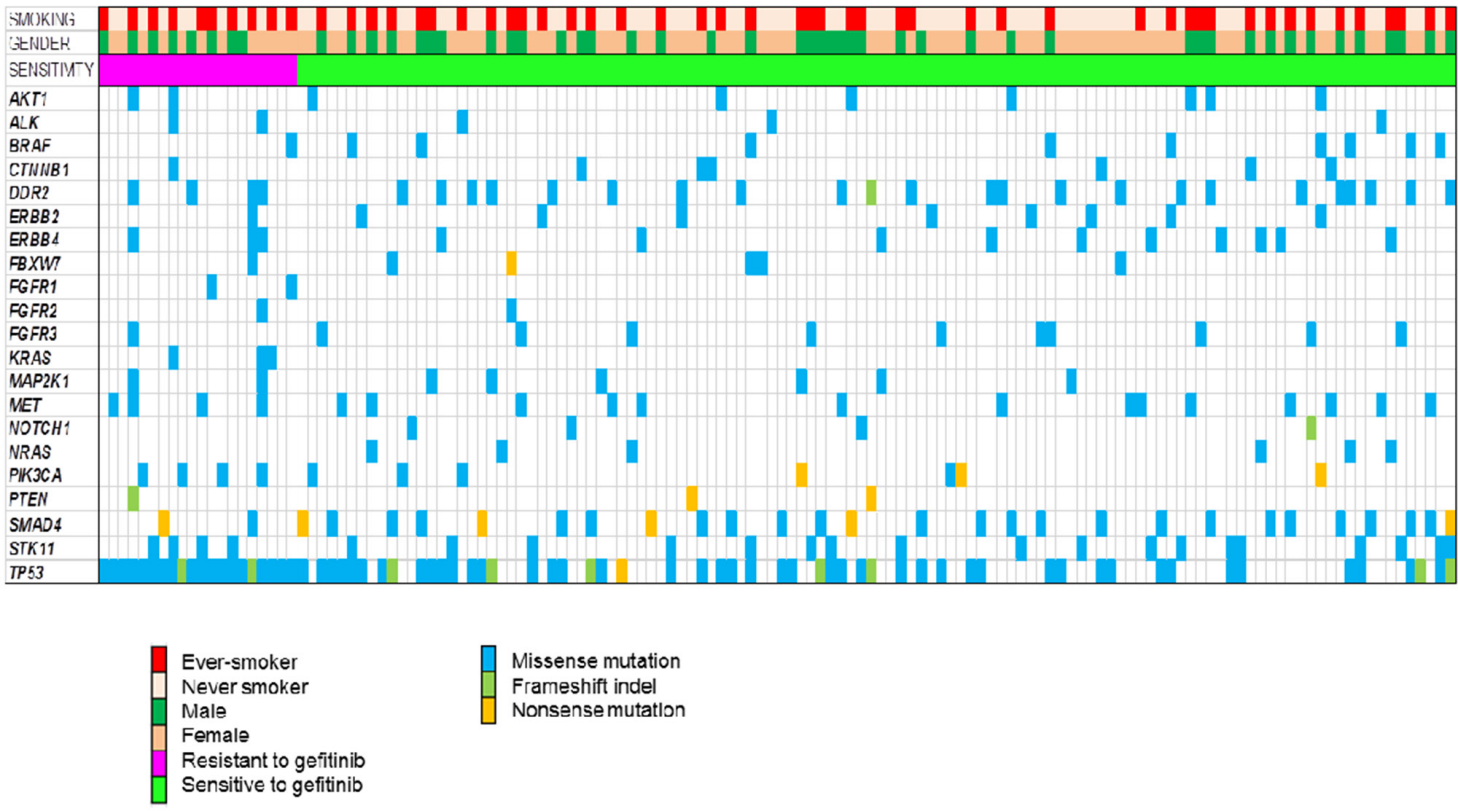
Landscape genomic profile of patients Samples are grouped by response to gefitinib.

The genomic landscape of 136 patients is depicted in Figure 2. A total of 543 somatic single-nucleotide variants (498 missense, 13 nonsense) and 32 frameshift insertions/deletions, with a median of 3 mutations per sample. All patients harbored EGFR mutations (not depicted in Figure 2), and *EGFR* mutations included deletions in exon 19 (n=75), L858R (n=65), 1 missense mutation (Thr751Pro) in exon 19, 1 *de novo* T790M gatekeeper mutation in exon 20, 1 missense mutation (L861Q) in exon 19, 2 missense mutation (G873R, K860I) in exon 21, and 1 missense mutation (N700S) in exon 18.

The median number of mutations per sample in non-responders was 4, whereas the median number of mutations per sample in responders was 3. The number of mutations was significantly greater in non-responders compared to responders (13.6% vs. 10.6%, *P* = 0.009). There were 4 smokers who concurrently harbored 6 to 9 mutations among non-responders whereas all responders had mutations range from 1 to 4. In non-responders, all patients harbored *TP53* mutations and the proportion of smokers was higher than in responders (50% vs. 37%, *P* = NS). Of note, *KRAS* mutations were identified in 3 patients among non-responders, which were previously not detected by conventional testing by direct sequencing method.

*TP53* was most commonly mutated (47%) in all patients. Mutations in *SMAD4* was also common (19%), as well as *DDR2* (16%), *PIK3CA* (15%), *STK11* (14%), and *BRAF* (7%). Recurrent mutations in *AKT1, ALK, CTNNB1, ERBB2, ERBB4, FGFR1-3, MAP2K1, MET, NOTCH1, NRAS*, and *PTEN* were also noted.

### Mechanisms of primary resistance to EGFR TKI

**Figure 3:**
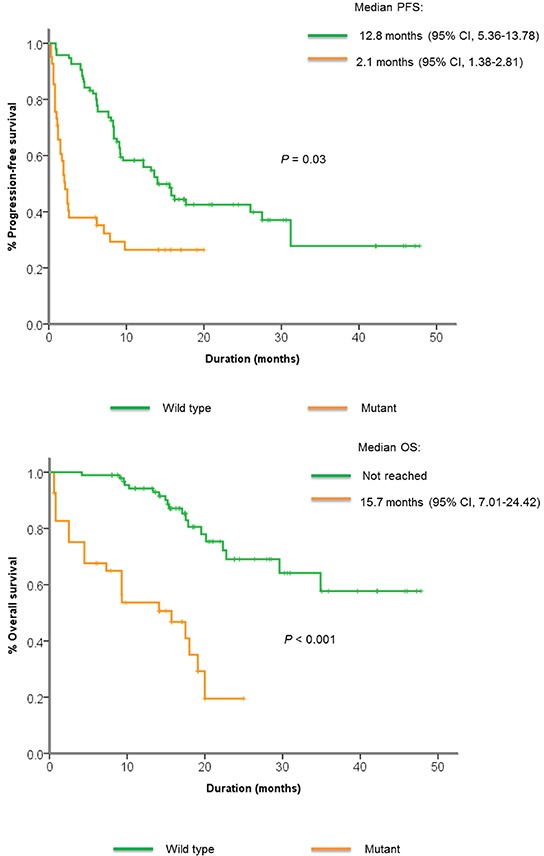
Kaplan-Meier curve showing **A.** median progression-free survival and **B.** median overall survival among patients with or without *PI3K/Akt/mTOR* pathway alterations.

Genomic mutations in the *PI3K/Akt/mTOR* pathway were commonly found in non-responders compared to responders (45% *vs*. 27%), although the difference was not significant. These mutations included missense mutations in *AKT1*, *PIK3CA*, *STK11*, nonsense mutations in *PIK3CA*, *PTEN*, and frameshift indels in *PTEN*. Patients with mutations in the *PI3K/Akt/mTOR* pathway had significantly shorter PFS and OS compared to those without (2.1 *vs*. 12.8 months, *P*=0.03, 15.7 *vs.* not reached, *P* < 0.001) (Figure 3A, 3B). *FGFR* 1-3 alterations were also more commonly found in non-responders compared to non-responders (20% *vs*. 8.3%, *P* = NS). All *KRAS* mutations were detected in non-responders (15% *vs.* 0%, *P* < 0.001), and *TP53* mutations were detected in 100% of non-responders compared to 39% in responders (*P* < 0.001). There were no significant differences in alterations between the patients who showed upfront resistance (n=5) *vs*. patients with stable disease < 4 months (n=15).

## DISCUSSION

Here, we described genomic alterations identified in patient tumors that showed primary resistance to gefitinib. We suggest that *PI3K/Akt/mTOR* pathway alterations may confer resistance to gefitinib.

Although EGFR TKI has undoubtedly revolutionized the treatment of *EGFR*-mutant lung cancer patients, but more investigation is necessary to elucidate mechanisms of primary resistance to EGFR TKI. Previous studies have assessed mechanisms of primary resistance to EGFR TKIs: alterations in *EGFR*-downstream genes, *EGFR* T790M mutation, *KRAS* mutation, *cMET* amplification and HGF overexpression [19–22]. However, most studies did not comprehensively examine the genomic alterations in association with treatment outcomes to EGFR TKI.

In our study, patients with pathway alterations in *PIK3CA/Akt/mTOR* pathway had significantly shorter PFS to EGFR TKI compared to those without. Genetic alterations in the *PIK3CA/Akt/mTOR* pathway may impact the response to EGFR TKI. This finding is consistent with the previous study that reported that mutations in *EGFR*-downstream genes such as *PIK3CA, AKT, PTEN* and *STK11* were associated with *de novo* resistance to EGFR TKI [8]. *PIK3CA* mutations were identified in 7% of our patients, and somatic missense mutations in helical or kinase domains of catalytic subunit encoded by the *PIK3CA* gene, which is known to increase the kinase activity of PIK3CA contributing to cellular transformation, were found in 2 out of 5 non-responder patients. We assume that activating *PIK3CA* mutations may have impaired response to EGFR TKI. Nine *AKT* mutations (5%) were identified in our patients and all mutations were located in pleckstrin homology domain. Of note, *AKT1* E17K mutation, previously known to activate PI3K pathway, was found in 1 non-responder which could explain the non-response to gefitinib [23]. *PTEN* is a tumor suppressor gene which negatively regulates the PI3K/Akt/mTOR pathway, and loss of PTEN was previously identified as a poor prognostic marker [24]. While 1 frameshift deletion (p.Glu242fs) has been noted in a non-responder, its functional impact is not yet known. Mutations in *STK11* were commonly identified (14%) and inactivation of *STK11* has been known to promote lung tumorigenesis and associated with worse survival outcome [25, 26]. Pathogenic *STK11* mutations (p.Phe354Leu) were recurrently identified in 3 non-responders whereas pathogenic mutations were not seen in responders. In patients with *PI3K/Akt/mTOR* pathway alterations, combination of PI3K inhibitor with gefitinib may be attempted to as a new therapeutic option.

Apart from *PIK3CA/Akt/mTOR* pathway alterations, *KRAS* mutations were identified exclusively in non-responders. *KRAS* mutations are known predictors of resistance to EGFR TKI [21], and interestingly, these mutations were previously not found with conventional sequencing. Possible reasons for the discordant results are low sensitivity of detection method and intratumoral heterogeneity. Genomic alterations with low allele frequencies lead to false-negative results on conventional sequencing and subclonal mutations may be heterogeneous according to biopsy sites [27, 28].

*TP53* was the most commonly altered gene in our study and this is consistent with findings from previous studies [29]. Of note, all non-responders had *TP53* mutations (frameshift deletion and missense mutations). Interestingly, it has been reported that *TP53* may enhance sensitivity to EGFR inhibitor, and loss of *TP53* may lead to resistance to EGFR inhibitor [30]. Four pathogenic mutations were identified among non-responders, and they may have led to functional loss of *TP53*, resulting in primary resistance to gefitinib.

Six uncommon *EGFR* mutations identified in our study were missense mutations in exon 18 (F712S, G721A) and 19 (A743S, P733L, L747F, N756D). Their oncogenicity and sensitivity to EGFR TKI have not been fully elucidated. Wu *et al*. observed an objective response rate of 48.4% and a median PFS of 5.0 months in patients with uncommon *EGFR* mutations [31]. In our study, two uncommon *EGFR* mutations were identified among non-responders and four were identified among responders. Prospective trials which examine the efficacy of EGFR inhibitors in patients with uncommon *EGFR* mutations are necessary.

Our study has a few limitations. The selected genes in this study may only explain a portion of mechanisms of resistance. Other genetic or epigenetic alterations that are not covered in the gene test may be missed out even if they promote resistance to EGFR TKI. However, most tumor samples were acquired from small biopsy samples and thus there were not enough tissue available for a more comprehensive sequencing. In addition, the number of patients analyzed was relatively small and 35 (25.7%) patients did not receive gefitinib as their first line of therapy, so data must be interpreted cautiously. Lastly, functional effects of resistant alterations were not assessed *in vitro*.

In conclusion, we note that more comprehensive genomic characterization of the tumor reveals alterations that may confer resistance to EGFR TKI in *EGFR*-mutant lung adenocarcinoma patients. This study highlights previously unappreciated genetic alterations, enabling further refinement in sub-classification for the improved personalization of lung cancer treatment.

## MATERIALS AND METHODS

### Study populations

A total of 152 patients with NSCLC harboring activating *EGFR* mutations were enrolled in a prospective trial of gefitinib between 2012 and 2015 at institutions in Korean Lung Cancer Consortium. Activating *EGFR* mutations were defined as mutations known to be associated with EGFR TKI sensitivity, including exon 19 deletion and L858R [14]. Patients with available archival tissue and those with measurable lesions at baseline were enrolled. This study was approved by the Institutional Review Board of Severance Hospital and the ethics committee. All patients provided written informed consent for study participation and genetic analysis.

### Data collection

Medical records and radiologic images of all patients were reviewed to evaluate demographic and clinicopathologic parameters, tumor response and progression-free survival (PFS) and overall survival (OS) using a predesigned data collection format. PFS was measured from the first day of treatment with EGFR TKI to tumor progression or death. OS was measured from the first date of treatment with EGFR TKI until the date of death. Patients were censored on December 7, 2015 if alive and progression-free. Never-smokers were defined as those with a lifetime smoking-dose less than 100 cigarettes.

### Tumor assessment

Response to gefitinib was assessed by a computed tomography scan performed at 4 weeks and then every 8 weeks thereafter in accordance with the Response Evaluation Criteria in Solid Tumors (RECIST) version 1.1 [15]. Primary resistance to EGFR TKIs was defined as followings: (1) the best response to EGFR TKI is progressive disease (PD) and (2) PFS to EGFR TKI is less than 4 months with stable disease (SD) as best response [8, 16].

### Genetic analysis

Tumor samples from 136 patients were available for genetic alterations. DNA was extracted from FFPE tumor samples using the QIAamp FFPE tissue kit (Qiagen, Antwerp, Belgium). The DNA obtained was quantified using the Qubit® fluorometer in combination with the Qubit dsDNA HS assay kit (Life Technologies, Gent, Belgium). For library construction, 10ng of DNA was amplified using the Colon and Lung Cancer panel (Ampliseq, Life Technologies). An amplicon library was generated for sequencing 1825 hotspot mutations in 22 genes including *AKT1* (NM_05163), *ALK* (NM_004304), *BRAF* (NM_004333), *CTNNB1* (NM_001904), *DDR2* (NM_001014796), *EGFR* (NM_005228), *ERBB2* (NM_004448), *ERBB4* (NM_005235), *FBXW7* (NM_033632), *FGFR1* (NM_023110), *FGFR2* (NM_022970), *FGFR3* (NM_000142), *KRAS* (NM_033360), *MAP2K1* (NM_002755), *MET* (NM_001127500), *NOTCH1* (NM_017617), *NRAS* (NM_002524), *PIK3CA* (NM_006218), *PTEN* (NM_000314), *SMAD4* (NM_005359), *STK11* (NM_000455), *TP53* (NM_000546). The amplicons were then digested, barcoded and amplified using the Ion Ampliseq™ Library kit 2.0 and Ion Xpress™ barcode adapters kit (Life technologies) according to the manufacturer's instructions. The library was quantified using the Qubit1 fluorometer and the Qubit1 dsDNA HS assay kit (Life technologies). 8pM of each library was multiplexed and clonally amplified on Ion sphere™ particles (ISP) by emulsion PCR performed on the Ion One Touch 2 instrument with the Ion PGM™ template OT2 200 kit (Life technologies) according to the manufacturer's instructions. Quality control was performed using the Ion Sphere™ Quality Control kit (Life Technologies) to ensure that 10–30% of template positive ISP were generated in the emulsion PCR. Finally, the template ISP were enriched, loaded on an Ion 316™ or on an Ion 318™ chip and sequenced on a PGM™ sequencer with the Ion PGM™ sequencing 200 kit v2 according to the manufacturer's instructions.

### Data analysis

The raw data were analyzed using the torrent suite software v3.6.2 (Life technologies). The coverage analysis was performed using the coverage analysis plug-in v3.6. Cases for which the number of mapped reads was <100000 and/or the average base coverage was <500x were considered as non-informative. Mutations were detected using the Variant Caller plug-in v3.6 with low stringency settings (Life Technologies). In the variant list obtained, each mutation was verified in the Integrative genome viewer (IGV) from the Broad Institute (http://www.broadinstitute.org/igv/). Only mutations reported in the COSMIC (Sanger Institute Catalogue of Somatic Mutations in Cancer) database (http://www.sanger.ac.uk/cosmic) were taken into account and silent or intronic mutations were not reported. Locis were further analyzed for functional prediction of amino acid changes using two different prediction algorithms (Provean and SIFT) [17, 18].

### Statistical analysis

OS and PFS were estimated using the Kaplan–Meier method. Differences between groups were compared by the log-rank test. Two-sided *P*-values < 0.05 were considered significant. All analyses were carried out using SPSS 20.0 (IBM SPSS Statistics, IBM Corp., Somers, NY).

## SUPPLEMENTARY FIGURES


