# Supplementary material for: Targeted sequencing identifies genetic alterations that confer primary resistance to EGFR tyrosine kinase inhibitor (Korean Lung Cancer Consortium)
Source: Oncotarget. 2016 Apr 21;7(24):36311–20. doi: 10.18632/oncotarget.8904 (PMC5095002; doi:10.18632/oncotarget.8904)
Supplement: Supplementary file 1 [file oncotarget-07-36311-s001.pdf]

SUPPLEMENTARY FIGURE

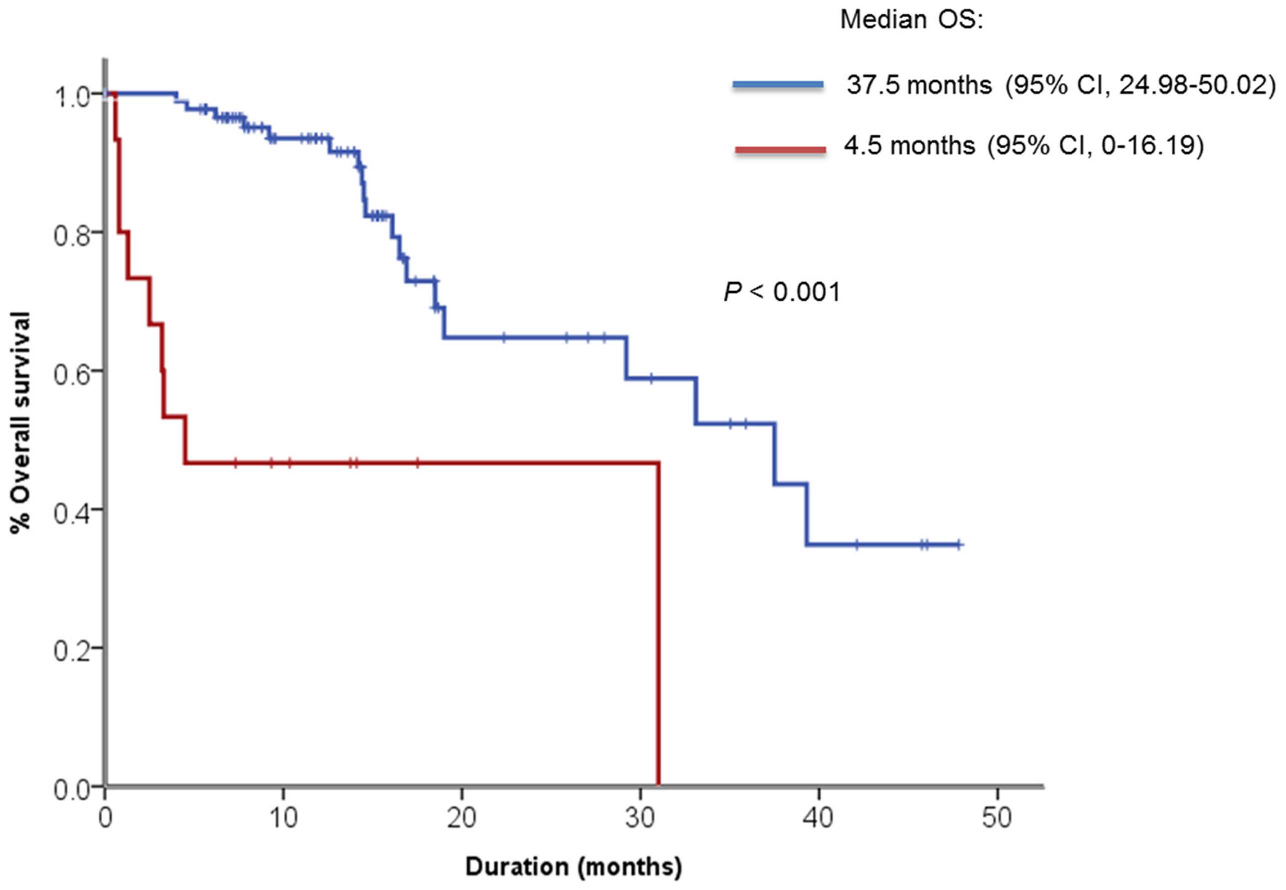

Supplementary Figure S1: Kaplan-Meier curve showing median overall survival among patients who received gefitinib as their first line of therapy.
